# Gradual transition of pyramidal cell types in the dorsal hippocampal area CA2b of the C57BL/6 mouse

**DOI:** 10.1038/s41598-025-04329-1

**Published:** 2025-08-11

**Authors:** Meike Fellenz, Rebecca Schneider, Sharif Jabra, Michael Rietsche, Dinko Smilovic, Mario Vuksic, David A. Slattery, Thomas Deller

**Affiliations:** 1 Institute of Clinical Neuroanatomy, Neuroscience Center, Goethe University Frankfurt, Theodor-Stern-Kai 7, 60590 Frankfurt am Main, Germany; 2 Croatian Institute for Brain Research - School of Medicine, University of Zagreb, Salata 12, 10 000 Zagreb, Croatia; 3 Department of Psychiatry, Psychosomatic Medicine and Psychotherapy, University Hospital, Goethe University Frankfurt, Heinrich-Hoffmann-Straße 10, 60528 Frankfurt am Main, Germany; 4 Present Address: Department of Diagnostic and Interventional Radiology, Marienhospital, 70199 Stuttgart, Germany

**Keywords:** PCP4, Thorny excrescence, Stratum lucidum, Mossy fiber, Intracellular injection, Electron microscopy, Neuroscience, Cellular neuroscience

## Abstract

Hippocampal area CA2 is important for social recognition memory. It has classically been defined as the region between areas CA3 and CA1 where pyramidal cells have larger cell bodies than CA1 neurons, but lack mossy fiber input and thorny excrescences (TEs) typical for CA3 neurons. Based on molecular signatures, the borders of area CA2 have been redefined, with area CA2b now covering parts of former area CA3a. Functional data suggest that CA2b is a mixture of CA3 and CA2 cells, yet information about the spatial distribution of these cells within CA2b remains vague. In the present study, we filled pyramidal cells in dorsal CA3-CA2-CA1, post-hoc identified CA2 cells using Purkinje Cell Protein 4 (PCP4), and analyzed their proximal apical spines using confocal and electron microscopy. We found that dorsal CA2b resembles an intermediate zone between CA3 and CA2a, where CA3 neurons with large TEs are gradually replaced by CA2 neurons. These CA2 neurons are heterogeneous in their scarce display of spines and larger spiny protrusions, rarely also carry TEs, and form synapses with mossy fibers. Thus, morphology needs to be combined with molecular markers to identify CA2 neurons in area CA2b with certainty.

**Supplementary Information:**

The online version contains supplementary material available at 10.1038/s41598-025-04329-1.

## Introduction

Area CA2 is a highly specialized region of the hippocampal formation important for social recognition and contextual fear memories^1,2^. It was first described by Lorente de Nó, who distinguished CA2 as a small group of neurons between Cajals hippocampal regio “superior” and “regio inferior” based on cell-anatomical criteria. CA2 neurons had larger cell bodies than CA1 neurons, but at the same time lacked mossy fiber input as well as thorny excrescences, the large complex spines typically found on proximal dendrites of CA3 pyramidal cells^3^. In recent years, however, the discovery of unique expression profiles, electrophysiological properties, and afferent connectivity of CA2 pyramidal cells^4–10^ proved that area CA2 extends beyond these borders. Newly established CA2 marker proteins like Purkinje Cell Protein 4 (PCP4)^6^, Regulator of G-protein Signaling 14 (RGS14)^11^, and Striatal Enriched Protein Tyrosine Phosphatase (STEP)^12^ now characterize CA2 based on its molecular signature. This redefined area CA2 is currently subdivided into CA2a, corresponding to de Nó’s classical CA2, and CA2b, which was formerly regarded as a distal part of CA3a (reviewed in^13^).

Of note, this shift in CA2 borders into former CA3 territory entails that in contrast to CA2a neurons, apical dendrites of CA2b neurons traverse the *stratum lucidum*, i.e. the layer where mossy fibers form large presynaptic contacts with postsynaptic thorny excrescences on dendrites of CA3 pyramidal cells. As the classically defined CA2 cells in CA2a lack mossy fiber input, this raises the question, which forms of contact molecularly defined CA2 cells in CA2b establish with mossy fibers. Electrophysiological studies show that both mature and newborn granule cells form direct, excitatory, monosynaptic connections with CA2 cells in CA2b^7,14^. Interestingly, analysis of mossy fiber terminals (MFTs) revealed that MFTs found in *stratum lucidum* of CA2b were considerably smaller and less numerous than the giant MFTs seen in CA3^7,9,14^, consistent with a much weaker excitatory response of CA2 neurons following optogenetic stimulation of MFTs expressing channelrhodopsin2-YFP^7,8^. This apparent input from mossy fibers contrasts with reports indicating that one of the defining features of classical CA2 neurons, i.e. the lack of thorny excrescences, also applies to molecularly-defined CA2 cells in CA2b. Proximal apical dendritic trees of murine CA2 cells in CA2b were described as aspiny or sparsely spiny^7,15^, and no thorny excrescences have to date been reported on identified CA2 cells.

Studies investigating electrophysiological properties of neurons within CA3 and CA2 complement these data and suggest that CA2b could be a heterogeneous zone where CA3 and CA2 cells intermingle: One study found spatial coding properties to differ between CA3 cells and CA2a cells, and the gradual transition of these properties takes place in a region corresponding to area CA2b^16^. Other studies examined the cellular morphologies of recorded and filled neurons and reported the presence of both neurons with thorny excrescences and athorny cells in an area corresponding to CA2b^8,17,18^. Thorny cells in this area were also functionally distinct from athorny cells in several electrophysiological parameters, among others displaying a higher input resistance and a more negative resting membrane potential^8,18^. Although these studies suggest that CA2b contains a mix of CA3 and CA2 cells, a systematic analysis of the spatial distribution and morphological characteristics of multiple pyramidal CA2 and non-CA2 cells in and around the molecular CA2 in the wildtype mouse hippocampus is missing.

To fill this gap and to resolve the open issues, we filled pyramidal cells along the dorsal CA3-CA2-CA1 axis in fixed brain sections of naïve, adult male and female wildtype C57BL/6 mice. By employing post-hoc immunolabeling with the CA2 marker PCP4, CA2 and non-CA2 cells were identified and their spatial distribution was mapped. Moreover, we closely examined their proximal apical dendrites to evaluate the types of spines formed within the different regions. Finally, immuno-EM was employed to demonstrate synaptic contacts between MFTs and PCP4-positive CA2 neurons.

## Results

**Fig. 1:**
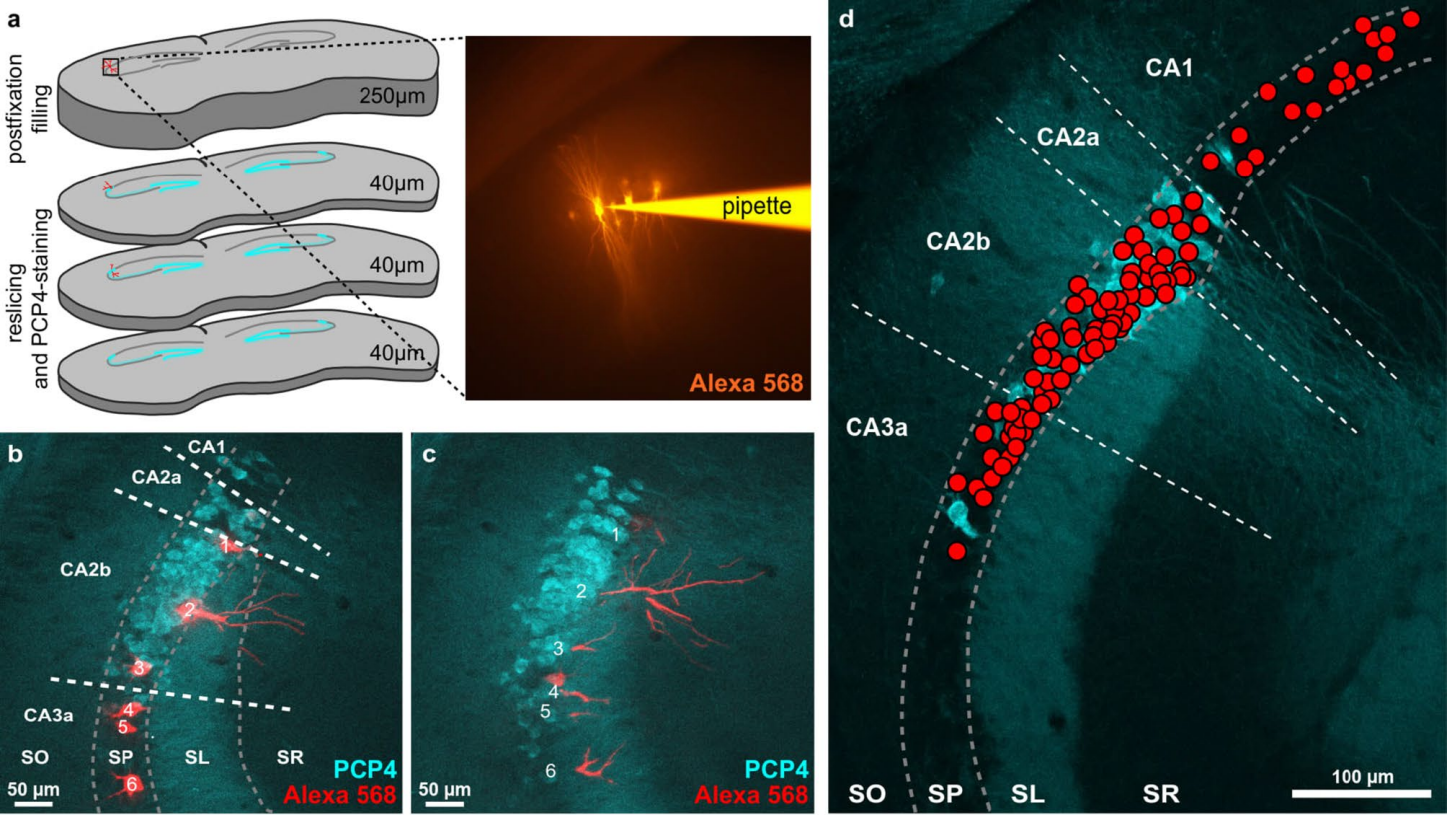
Identification of PCP4-positive cells in sections of brain slices filled iontophoretically with an Alexa dye. (**a**) Neurons along the CA3-CA2-CA1 axis of the dorsal hippocampus were iontophoretically injected with a fluorescent Alexa dye (Alexa 568 or Alexa 488) in 250 μm thick fixed brain sections. Sections were subsequently resliced to 40 μm to facilitate the following PCP4 staining. (**b**) Filled, resliced and stained sections were imaged and maximum-Z-projections were used to define the borders of the CA regions using the PCP4 staining in the section containing the cell somata. Cell bodies of filled cells were then numbered and identified as PCP4-positive or PCP4-negative in higher-magnification images. (**c**) If proximal apical dendrites were not on the same section as the cell bodies, dendrites on adjacent sections were assigned to their respective cell bodies on the basis of their location within the section. (**d**) Relative positions of all 90 evaluated filled neurons from a total of five male and six female C57BL/6 mice. SO - *stratum oriens*; SP - *stratum pyramidale*; SL - *stratum lucidum*; SR - *stratum radiatum*; CA - *cornu ammonis*.

For the purpose of this study, we re-imaged sections originally prepared for a previously published report^19^. There, we had randomly injected discrete pyramidal cells in the dorsal hippocampus in fixed brain slices of male and female C57BL/6 mice with Alexa 488 or Alexa 568, and had post-hoc stained for the molecular CA2 marker PCP4 to identify CA2 cells (Fig. 1a, b, c). We were able to evaluate PCP4 expression, proximal apical morphology, soma area, as well as relative location, of 90 filled pyramidal cells in the dorsal hippocampus from a total of five male and six female mice ranging from distal CA3 (CA3a) to the proximal CA1 (Fig. 1d). As established in previous publications^7,12^, the PCP4 immunostaining labeled the classical CA2 region, which lacks mossy fiber innervation (CA2a), as well as an abundance of cells proximal to CA2a (CA2b), with apical dendrites traversing *stratum lucidum*. Few scattered PCP4-positive cells could be detected outside this region in distal CA3 and proximal CA1 (cf. Figure 1b, d). We accordingly first used the PCP4 staining to set the approximate boundaries of area CA2, and to further divide CA2 into CA2a and CA2b, with the distal tip of *stratum lucidum* marking the border between the CA2 subfields (Fig. 1b, c, d, and Methods). Higher magnification images of the respective neurons were then used to identify the filled cell bodies as PCP4-positive (putative CA2 cell) or PCP4-negative (non-CA2 cell).

### PCP4-positive neurons in CA2b show large spiny protrusions, and rarely also thorny excrescences, in *stratumlucidum*

**Fig. 2:**
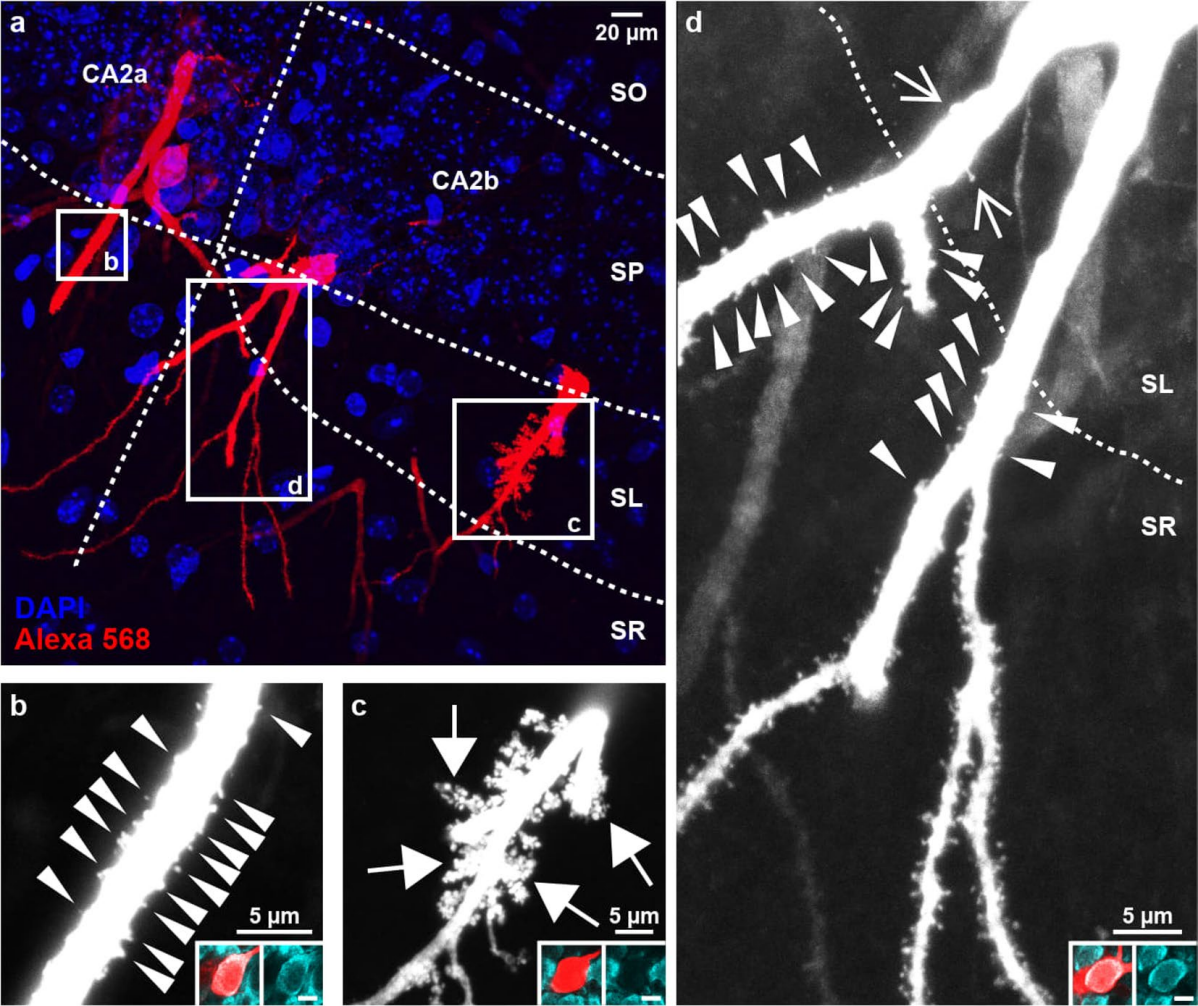
Spine morphologies of apical dendrites of neurons in areas CA2a and CA2b. (**a**) Overview maximum-Z-projection of three injected neurons in CA2. White boxes indicate the regions magnified in b-d. (**b**) Proximal apical dendrite of a PCP4-positive neuron in CA2a. Note the presence of classical spines in SR even in the most proximal parts of the main trunk (arrowheads). (**c**) Proximal apical dendrite of a PCP4-negative neuron in CA2b. The main apical trunk is densely covered with large and elaborate thorny excrescences (filled arrows) in SL, indicating mossy fiber contact. (**d**) Proximal apical dendrite of a PCP4-positive neuron in CA2b. The main trunk crossing SL is mostly devoid of spines, only two spinous structures are visible (open arrows). Once the dendrite enters SR, it is richly decorated with classical spines (arrowheads). Image was stitched together from maximum-Z-projections of two partially overlapping image stacks. b-d insets: cell somata belonging to the respective dendrites were identified in the adjacent section (red: Alexa 568, cyan: PCP4). Scale bars: 10 μm. SO *– stratum oriens*; SP – *stratum pyramidale*; SL – *stratum lucidum*; SR *– stratum radiatum*.

**Fig. 3:**
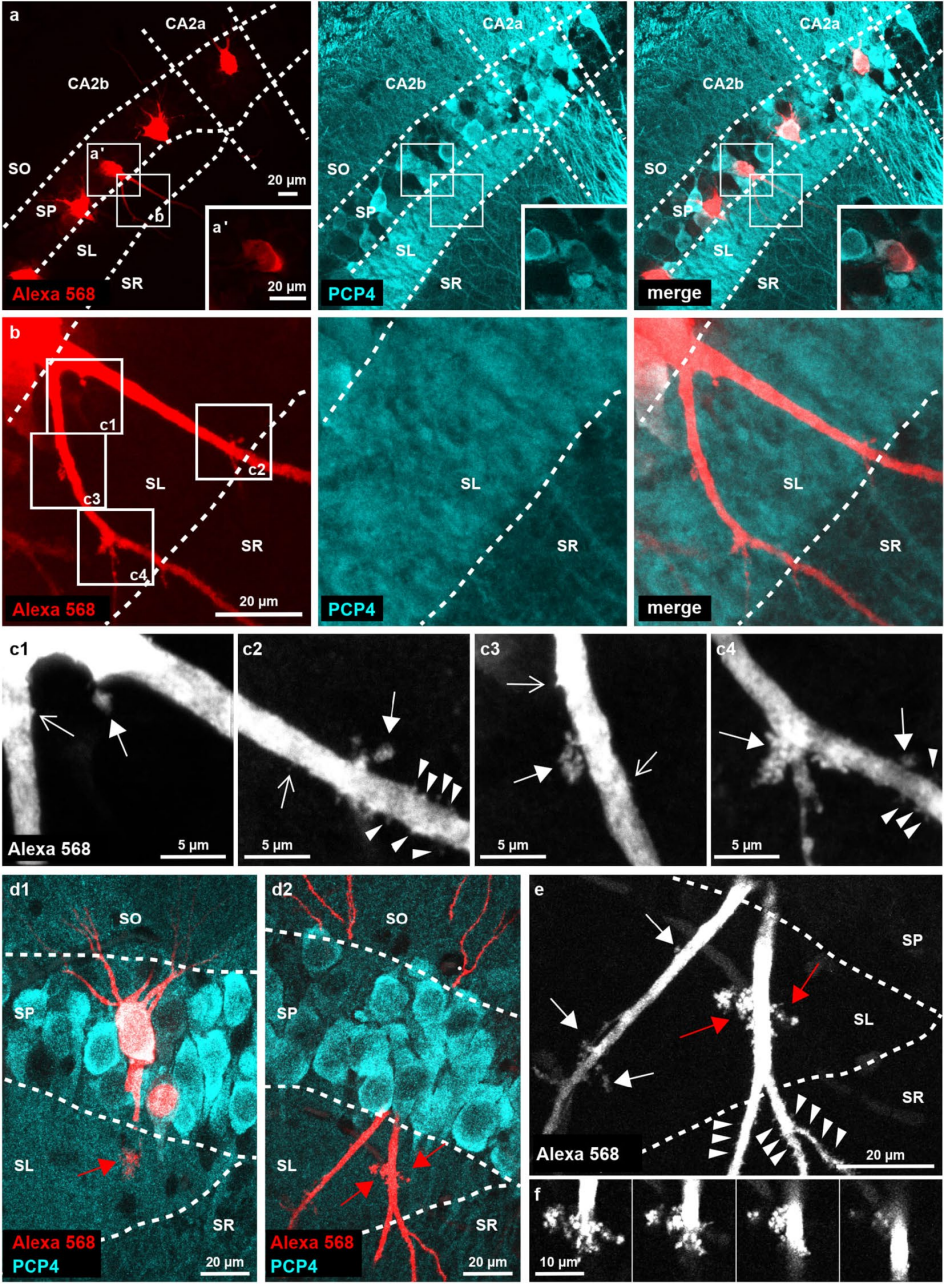
PCP4-positive neurons in CA2b show large spiny protrusions, and rarely also thorny excrescences, in *stratum lucidum*. (**a**) Overview maximum-Z-projection of the injected CA2 region showing the relative position of a PCP4-positive neuron in CA2b. (**a’**) The cell body is positive for PCP4. White boxes indicate the regions magnified in **a’** and **b**. (**b**) Magnification of the proximal apical tree. White boxes indicate the regions further magnified in **c1-4**. (**c**) Spiny protrusions with large heads (filled arrow) are visible alongside some classical spines (open arrow) in SL (**c1**,** c2**). Bigger spiny protrusions with a more lobular structure reminiscent of complex spines are also visible (**c3**,** c4**; filled arrows). Note the emergence of classical spines (arrowheads) as the apical tree enters SR (**c2**,** c4**). (**d**) Two adjacent sections showing a PCP4-positive neuron in distal CA2b carrying several large thorny excrescences (TEs). For visualization purposes, the maximum-Z-projection of the labeled neuron (Alexa 568) is overlayed with only the one PCP4 channel image plane of the stack that contains the cell body of the labeled neuron. The cell body (**d1**) is clearly PCP4-positive. The proximal apical tree on the adjacent reslice (**d2**) displays the excrescences (red filled arrows), part of which can also be seen in **d1**. (**e**) Magnification of **d2**. The TEs (red filled arrows) as well as several larger spinous protrusions (white filled arrows) are clearly visible. The dendrites are densely covered with classical spines upon entering SR (arrows). (**f**) Magnification of the large TEs visible in **e**, followed through four consecutive image planes of the stack. SO *– stratum oriens*; SP – *stratum pyramidale*; SL – *stratum lucidum*; SR *– stratum radiatum*; CA – *cornu ammonis*.

In a first step, we qualitatively investigated the appearance of spiny protrusions on the proximal apical dendrites of cells in area CA2. The thick proximal apical dendrites of PCP4-positive cells in CA2a, which are not in contact with mossy fibers, were richly decorated with classical spines as soon as they emerged from the pyramidal cell layer and entered *stratum radiatum* (Fig. 2a, b). This distinguishes them from CA1 cells, whose proximal stretches of thick primary apical dendrite are mostly aspiny or only sparsely spiny^20^. The apical dendrites of all PCP4-negative cells in CA2b and CA3 *stratum lucidum* were densely covered with elaborate thorny excrescences, typical for mossy fiber input onto CA3 cells (Fig. 2c). In contrast, the proximal apical trunks of PCP4-positive cells in CA2b displayed hardly any spiny structures in *stratum lucidum*, whereas the dendrites were richly decorated with classical spines as soon as they entered *stratum radiatum* (Fig. 2d). While the proximal apical tree of most PCP4-positive cells seemed to be devoid of any discernible spines, or only showed a few normal-sized spines in *stratum lucidum* (Fig. 2d), some neurons displayed considerably larger protrusions that were scarcely scattered along the proximal apical dendrite (Fig. 3a, b). These protrusions were mostly simple in shape, showing one large bulbous head (Fig. 3c1, c2). Yet, they sometimes also showed a more lobular structure reminiscent of small complex spines (Fig. 3c3, c4). Three out of a total of 28 PCP4-positive neurons in CA2b carried one or several large thorny excrescence(s) (Fig. 3 d1, d2, e, f) that were comparable in complexity to the thorny excrescences found on CA3 neurons.

**Fig. 4:**
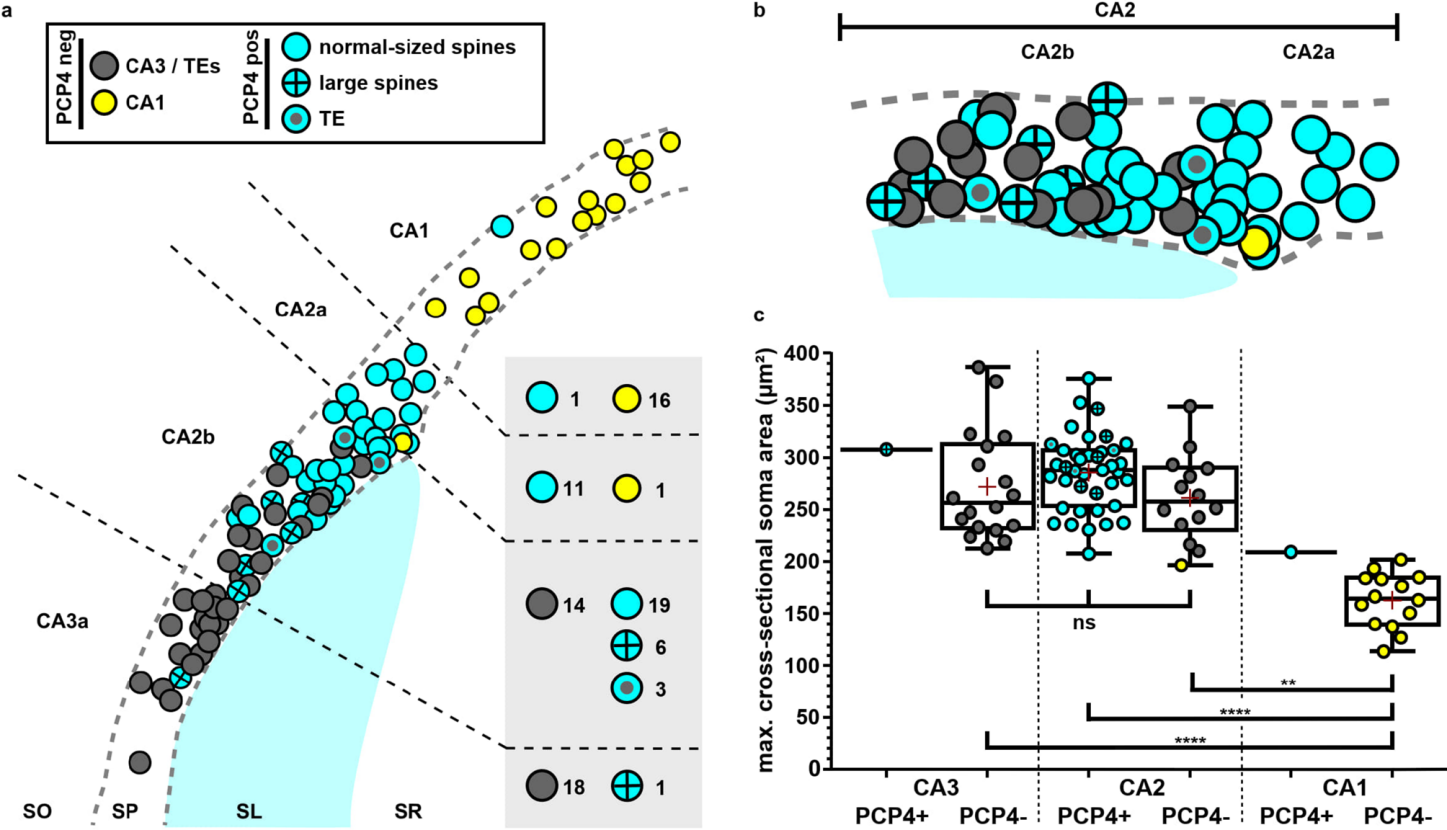
CA3 cells are gradually replaced by CA2 cells in CA2b. (**a**) Schematic overview of the relative distribution of PCP4-positive and PCP4-negative cells. Cells were further divided into the following categories depending on soma size and morphology of spines on the proximal apical trunk: PCP4-negative | TEs | large soma (gray); PCP4-negative | small soma (yellow); PCP4-positive | normal-sized spines | large soma (cyan); PCP4-positive | large spines | large soma (cyan with cross); PCP4-positive | TE(s) | large soma (cyan with dot). PCP4-negative cells with a large soma were assumed to be CA3 cells, PCP4-negative neurons with a small soma were assumed to be CA1 cells, PCP4-positive neurons were assumed to be CA2 cells. (**b**) Close-up of area CA2. Color coding as in **a**. (**c**) Soma sizes of the neurons depicted in **a**. Color coding as in **a**. Somata of neurons in area CA3 and area CA2 are comparable in size, and are significantly larger than those of CA1 neurons. CA3/PCP4(-): 271.7 ± 52.06 μm², *n* = 18 cells, CA2/PCP4(+): 285.8 ± 35.95 μm², *n* = 39 cells, CA2/PCP4(-): 259.4 ± 42.24 μm², *n* = 14 cells, CA1/PCP4(-): 155.6 ± 27.39 μm², *n* = 14 cells (mean ± s.d.). Kruskal-Wallis test: H(4) = 38.69, *P* < 0.0001. Post-hoc Dunn’s multiple comparison test: CA3/PCP4(-) vs. CA2/PCP4(+): ns; CA3/PCP4(-) vs. CA2/PCP4(-): ns; CA2/PCP4(-) vs. CA2/PCP4(+): ns; CA1/PCP4(-) vs. CA3/PCP4(-): *P* < 0.0001, ****; CA1/PCP4(-) vs. CA2/PCP4(-): *P* = 0.0017, **; CA1/PCP4(-) vs. CA2/PCP4(+): *P* < 0.0001, ****. Red cross indicates the mean. The two single CA3/PCP4(+) and CA1/PCP4(+) cells are presented in the graph for the sake of completeness, but were not included in the statistical analysis. Soma sizes of one CA2/PCP4(-) cell and two CA1/PCP4(-) cells shown in **a** could not be measured. TE – *thorny excrescence*; SO *– stratum oriens*; SP – *stratum pyramidale*; SL – *stratum lucidum*; SR *– stratum radiatum*; CA – *cornu ammonis.*

By chance, we also labeled two PCP4-positive neurons outside of area CA2, one of them in CA3a, and one in proximal CA1. Interestingly, the apical dendrite of the PCP4-positive neuron in CA3a was again mostly aspiny with the occasional spine-like protrusion, while the PCP4-negative neurons that were labeled in its vicinity were richly decorated with thorny excrescences (see Supplementary Fig. S1 online). The PCP4-positive neuron in CA1 had a bifurcating proximal apical dendrite (not shown), which is common in CA2 neurons, and a larger soma size than the neighboring CA1 neurons (cf. Figure 4c), indicating that this cell is not a PCP4-expressing CA1 cell, but a pyramidal neuron with CA2-like characteristics.

### CA3 cells are gradually replaced by CA2 cells in CA2b

After this qualitative analysis, we aggregated the data obtained from all 90 CA2 and non-CA2 neurons (34 cells from five males and 56 cells from six females) to create an overview map of their relative position across the proximodistal CA3-CA2-CA1 axis, along with the different spine structures we observed on the proximal apical trunk of those neurons.

As expected, randomly filled neurons in both distal CA3 and proximal CA1 were PCP4-negative, with the exception of one PCP4-positive cell in CA3a and CA1, respectively (Fig. 4a). Thorny excrescences of varying shape and size were present on proximal apical dendrites of all filled PCP4-negative cells in CA3a, therefore likely representing classical CA3 cells. Cell soma sizes of PCP4-negative cells in CA1 were significantly smaller than the soma sizes of PCP4-negative cells in CA3a (Fig. 4c), typical for CA1 cells. The majority of labeled cells in CA2b presented as a mixture of PCP4-negative cells with a large soma and thorny excrescences along the proximal apical dendrite (CA3 cells), and PCP4-positive cells with a large soma, but lacking such elaborate specializations (CA2 cells). As the number of CA3 cells gradually decreased from proximal to distal CA2b, CA2 cells increased in number (Fig. 4a, b).

The large spines we observed on the apical tree of some PCP4-positive neurons in CA2b were clearly distinct from the normal-sized spines found on *stratum lucidum* dendrites. In an attempt to define these large spines, we acquired additional high-resolution images of the dendritic tree in proximal *stratum radiatum* of three PCP4-positive CA2b neurons from three different animals, and measured spine length and maximal head width of a total of 465 spines to obtain a distribution of spine sizes in *stratum radiatum*. In addition, we measured visible spines on *stratum lucidum* apical dendrites of PCP4-positive neurons in CA2b (see Supplementary Figure S2 online). In *stratum radiatum*, spines with a head wider than 1.0 μm were not found. Also, spines that exceeded a length of 1.5 μm were rare, and were either filopodia-like structures with no clear head, or thin spines with a head diameter less than 0.7 μm. While most spines in *stratum lucidum* followed a comparable distribution, large spines formed a clearly distinct, additional population (Supplementary Figure S2 online). Based on this comparison, we defined a large spine as either (I) longer than 1.5 μm and with a bulbous head > 0.8 μm in width, or (II) with a maximal head width > 1.2 μm, irrespective of length.

Applying those criteria, we divided PCP4-expressing cells in CA2b into three groups: (1) cells that only displayed normal-sized spines, (2) cells that in addition displayed at least one large spine, and (3) cells with at least one large, lobular thorny excrescence in *stratum lucidum*. Indeed, the majority of PCP4-positive neurons in CA2b had a proximal apical trunk displaying only a few normal-sized spines (19/28), and only a subset of cells also displayed large spines in addition to the normal-sized ones (6/28). Cells with large spines appeared to be more frequent in the proximal half of CA2b. Three filled PCP4-positive cells in CA2b in addition carried one or several complex spines comparable to a thorny excrescence, displaying a stem with multiple bulbous heads (3/28, cf. Figure 3e, f).

CA2a was populated with PCP4-positive CA2 cells (11) that displayed classical spines even in the most proximal parts of the apical trunk (Fig. 4b). Cell soma sizes of PCP4-positive and PCP4-negative cells in CA3a and CA2 were comparable (Fig. 4c). One labeled cell in CA2a, situated superficially at the CA2a/CA2b border, was found to be PCP4-negative. As its cell body was small (cf. yellow dot in Fig. 4c), and the apical trunk displayed radial obliques covered in normal spines, this cell likely represents an intermingling CA1 cell.

As we previously reported sex differences in the spine densities of male and female mice in identified CA2 neurons^19^, we also investigated the distribution and appearance of pyramidal cells of males and females separately. PCP4-positive cells with thorny excrescences, PCP4-positive cells with large spines, and intermingling of CA3 and CA2 cells in CA2b were observable in both sexes (see Supplementary Fig. S3 online).

### Mossy fibers contact spiny protrusions of PCP4-positive neurons in CA2b

While electrophysiological studies have proven the existence of direct, excitatory connections of dentate granule cells with CA2 neurons in CA2b^7,14^, the morphological nature of these contacts is less clear. Immunohistochemical data imply contact of MFTs with RGS14-positive neurons^7^, however, light microscopy and co-immunostainings lack the resolution to provide sufficient detail. In a last experiment, we therefore determined on the subcellular level whether the spines and larger protrusions we observed on PCP4-positive neurons were indeed sites of MFT contact. To this end, we performed immuno-EM on a different set of brain sections, and used PCP4 pre-embedding immunohistochemistry to identify CA2 dendrites and to perform a qualitative analysis of the spiny synapses on the proximal apical dendrites of the neurons.

**Fig. 5:**
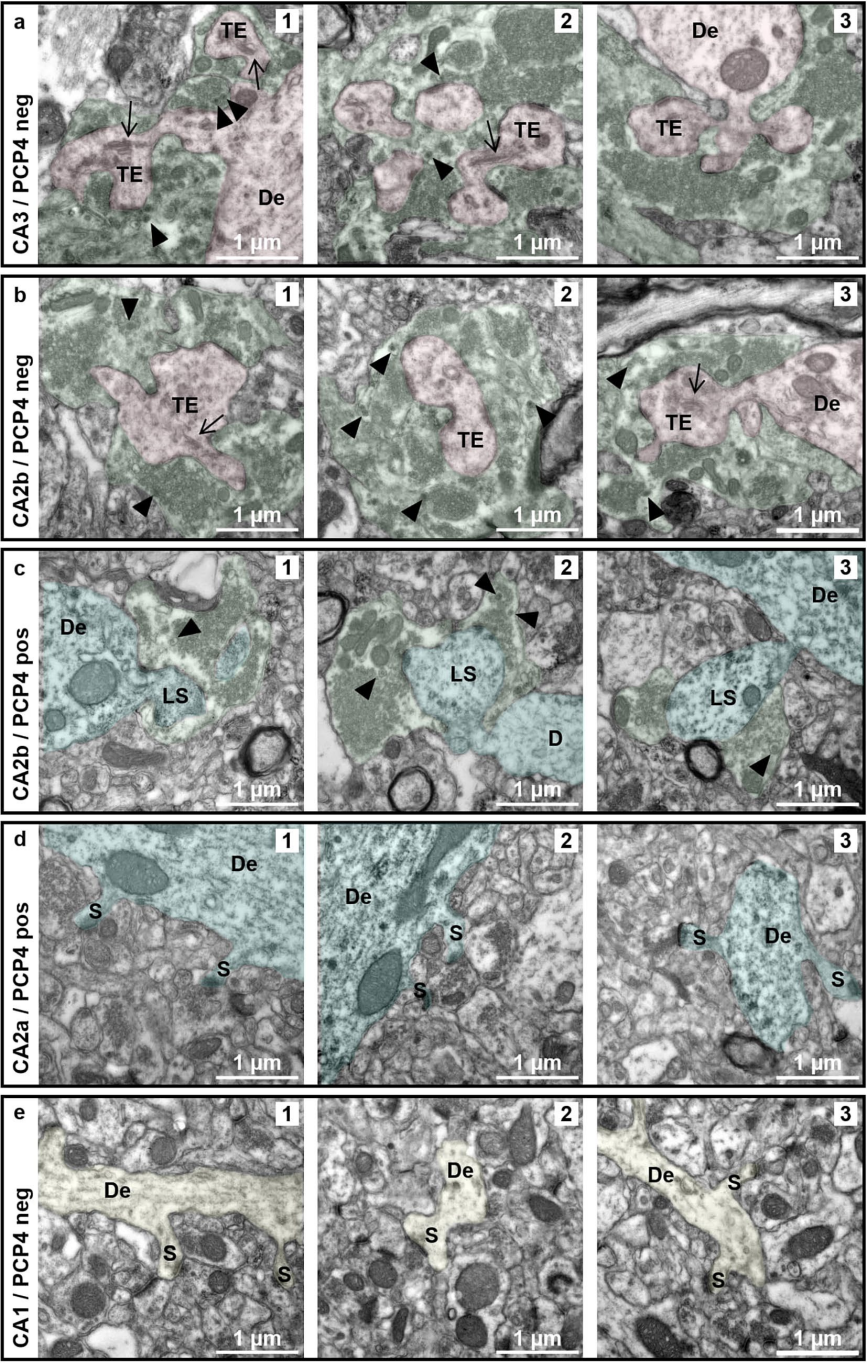
Mossy fibers contact spiny protrusions of PCP4-positive neurons in CA2b. EM images of sections stained for PCP4 (dark precipitate) to distinguish PCP4-positive from PCP4-negative neurons. (**a**,** b**) Large and elaborate thorny excrescences are readily found on PCP4-negative neurons in CA3 (red, **a1-3**) and CA2b (red, **b1-3**). They often contain a spine apparatus (arrow), and are enwrapped by mossy fiber terminals (MFTs, green). Note the presence of dense core vesicles (arrowheads) typical for this type of presynapse. (**c**) PCP4-positive neurons in CA2b (blue) show larger spines that are in contact with MFTs (green). The incidence of these spines is low. MFTs appear to be smaller than the ones contacting the thorny excrescences of PCP-positive neurons. (**d**) Dendrites of PCP4-positive neurons in CA2a (blue) display classical spines. Note that spines are also present on the proximal, larger-caliber dendrites (**d1**,** d2**). (**e**) Dendrites of PCP4-negative neurons in CA1 (yellow) display classical spines. Spines are mostly found on smaller-caliber dendrites, presumably radial obliques. De - *dendrite*; LS - *large spine*; S - *spine*; TE - *thorny excrescence*; arrow: *spine apparatus*; arrowhead: *dense core vesicle*.

The MFT was identified on the EM level using morphological criteria characteristic for this unique synapse: large size, multiple active zones, presence of a large pool of vesicles which are often grouped, and the presence of dense core vesicles^21^. PCP4-negative thorny excrescences contacted by MFTs identified by those criteria could readily be detected in CA3a (Fig. 5a) and proximal CA2b (Fig. 5b), reflecting the rich decoration of PCP4-negative neurons with excrescences as also seen in the light microscope. Given that the spiny protrusions on PCP4-positive neurons in CA2b are only sparsely scattered along the proximal dendrites of PCP4-positive neurons (cf. Figures 2d and 3b and e; Supplementary Fig. S1), we had to search several tissue blocks to find examples of them at the EM level. The spines we found presented with large heads and multiple active zones reminiscent of thorny excrescences, but lacked their elaborate lobular structure (Fig. 5c), consistent with the often mushroom-like shapes of the larger protrusions observed at the light microscopic level. The presynapses contacting those spines could be identified as MFTs, as they contained dense core vesicles and grouped synaptic vesicles typical for this type of presynapse. Although not quantified due to their low incidence, the terminals contacting these spines appeared to be smaller than the terminals enwrapping thorny excrescences of PCP4-negative neurons in CA3 and CA2b.

PCP4-positive neurons in CA2a displayed classical spines with normal size and shape (Fig. 5d), as did PCP4-negative neurons in CA1 (Fig. 5e). However, while spines could mostly be seen along dendrites with a smaller caliber in CA1, likely reflecting spines on radial obliques, such spines were also frequently found on primary apical dendrites with a larger diameter in CA2a, consistent with our findings at the light microscopic level (cf. Figure 2b).

## Discussion

In the present study, we have systematically analyzed the distribution of pyramidal cell types in areas CA2a and CA2b of the adult mouse hippocampus using dye-filling of pyramidal cells, post-hoc identification of CA2 neurons with PCP4, and confocal and electron microscopy. In agreement with others, we provide robust evidence that CA2b is a mixture of CA3 and CA2 cells^8,16–18^. Our study revealed that this mixture is not random, but rather represents a transition zone in which CA3 cells with large thorny excrescences are gradually replaced by CA2 cells.

The apical dendrites of most of the CA2b cells were found to be aspiny or sparsely spiny, as well as athorny, in *stratum lucidum*. However, closer examination of the CA2 dendrites in this subfield revealed that a fraction of CA2 cells also displayed larger protrusions. Surprisingly, and against the prevailing opinion that CA2 cells are all athorny, we did also detect PCP4-positive CA2 cells with large complex spines, although they were rare (cf. Figure 4). In addition, while heterogeneity of cells at the proximal border of CA2b was as expected^8,16–18^, we were surprised to still find PCP4-negative CA3 cells with thorny excrescences in the most distal parts of CA2b. These findings entail important implications for future studies: First, it implies that the absence of thorny excrescences is not a defining feature of CA2 cells and should not be used as the sole marker to distinguish CA3 cells from CA2 cells. Second, it shows that CA2 identity of a cell cannot be assumed based on its relative location within CA2b, even in its most distal parts.

Our finding of the presence of larger protrusions on CA2b neurons is similar to what has been reported for CA2 pyramidal cells in the guinea pig. In this earlier publication, proximal apical trunks of CA2 neurons were either aspiny or displayed sparse spines that were larger than the normal-sized spines present on the higher-order dendrites in *stratum radiatum*^22^. Thus, the larger-than-normal feature of spines on CA2b cells seems to be conserved across the two rodent species. Interestingly, a recent study conducted in mice also reported the presence of thorny excrescences in a low number of labeled neurons located in area CA2 within 200 μm of the distal-most tip of *stratum lucidum* towards CA3^9^. The neurons were labeled using a non-cell-type-specific GCaMP6f-AAV5 that had been stereotactically injected into area CA2, yet, no pos-thoc staining for marker proteins was employed to further confirm CA2 identity of the cells in question. As discussed, it can therefore not be ruled out that some of the cells bearing thorny excrescences detected in that region were not CA2 cells, but CA3 cells. However, both those findings and ours certainly necessitate future investigations, using cell-type-specific labeling of CA2 and/or CA3 neurons together with immunohistochemical CA2 markers, to further characterize the incidence and properties of these thorny excrescence-bearing CA2 neurons.

Previous studies have compared the overlap of different marker proteins used to delineate area CA2^7,9^, and found that the most commonly used CA2 markers PCP4, RGS14 and STEP overlap to a very high degree, especially in the dorsal hippocampus. The differences that were observed were mainly in the extent of labeling in and beyond the border areas towards CA3 and CA1^9^. Radzicki et al. reported that they observed no evidence of single cells that were either RGS14+/PCP4- or PCP4+/RGS14- in the zone that was reliably labeled by both markers in overview images, yet acknowledged that such cells might be found if higher-magnification images were examined. Therefore, it is possible that cells we identified as CA2 neurons using PCP4 staining might not express another marker protein. Exploring the heterogeneity of molecular profiles of single cells in area CA2 and correlating these profiles with cellular morphology and function will be an interesting avenue for future studies. Extending our work to single-cell identification of CA2 neurons in combination with different CA2 markers could, for example, shed light on the question whether the rare thorny excrescence-bearing CA2 cells differ from other cells in that they express some CA2 markers, but not others. 

Due to our methodology of slice preparation, our study can provide no further information regarding total neuronal morphology, as reslicing before immunostaining cuts the dendritic tree. A recent study, however, examined total neuronal morphologies from a large number of identified CA2 cells in mice^23^, and could show that cells can be divided into distinct morphological subtypes, similar to what has been previously described for the guinea pig^22^. Unfortunately, spine morphologies were not included in the analysis. In future studies, it might be of interest to see if different subtypes in CA2b also display different numbers or types of spiny protrusions on their proximal apical tree. Heterogeneity of cell types has not only been described around the CA3/CA2b border, but also around the CA1/CA2a border, where CA1 cells (identified either by morphological characteristics alone or by additional marker immunohistochemistry) were found to be intermingled with CA2 cells in rats^17,24^. While there was only one filled PCP4-negative cell with CA1-like characteristics in our sample, the total number of cells filled around this border was small and, thus, we cannot exclude that CA1 cells are more abundant at this border.

Investigating cellular heterogeneity in CA3 in mice, Hunt et al. found that pyramidal cells divide into a burst-firing and a regular-spiking group following current injections at or just above the rheobase^25^. Surprisingly, while the regular-spiking neurons all carried thorny excrescences, the burst-firing cells were devoid of thorny excrescences and were also PCP4-negative and VGLUT2 positive, identifying them as excitatory CA3 pyramidal cells. They described these cells to be present in deep CA3b and CA3a, and becoming more frequent towards CA2, although the total numbers were low. In contrast to the findings by Hunt et al., another recent electrophysiology study by Raus Balind et al. in rats also identified burst-firing cells in deep CA3, which reached as far as CA3c^26^. Prompted by the results from Hunt et al., they also checked for the presence of thorny excrescences, and found at least some excrescence-like “lobular” structures on all of those burst-firing cells, closely resembling the larger spiny protrusions found on PCP4-positive cells in our study. However, they did not do a post-hoc PCP4 stain, so it remains unclear if some of these cells might have been CA2 cells. While we found no PCP4-negative cells without thorny excrescences in CA3, it is possible that this cell type went undetected in our sample due to its scarcity and its usually deep location. The one athorny cell we did detect in CA3, however, was identified as one of the scattered PCP4-positive neurons that can be found outside of molecular CA2^7,12^. The athorny nature of these PCP4-positive cells in CA3a was also confirmed in a recent electrophysiological study^15^, where, despite their anatomical location, their electrophysiological properties were found to be more similar to CA2 cells than to CA3 cells^15^.

The presence of direct monosynaptic contacts between dentate granule cells and PCP4-positive neurons has been established on the electrophysiological level^7,14^, and fluorescent double stainings have suggested contacts between MFTs and both spiny protrusions and shafts of CA2 neurons^7^. Yet, light microscopy is limited in resolution, so we utilized pre-embedding immuno-EM to identify PCP4-positive neurons and to verify and further examine these contacts at the ultrastructural level. We could reveal the presence of direct contacts of MFTs with spines and larger spiny protrusions on proximal apical dendrites of PCP4-positive neurons using morphological criteria, i.e. the contacting presynaptic terminals were large, filled with numerous and occasionally clustered synaptic vesicles, and contained dense core vesicles. However, we cannot make a definite statement about the presence of MFTs synapsing onto shafts of those dendrites. While we occasionally did observe asymmetric active zones along the shafts of PCP4-positive neurons, there was no definite way of telling whether the contacting pre-synapse belonged to a mossy fiber with our methods, as they were small in size, with only a small number of vesicles, and no dense core vesicles were present. Likewise, small MFTs – which are more common in the distal part of CA2b - might be missed using only morphological criteria. More extensive EM studies, using pre-embedding co-immunohistochemistry for a CA2 marker and an MFT marker, along with silver- or gold-grains of different sizes to distinguish both stainings, may resolve this issue.

**Fig. 6:**
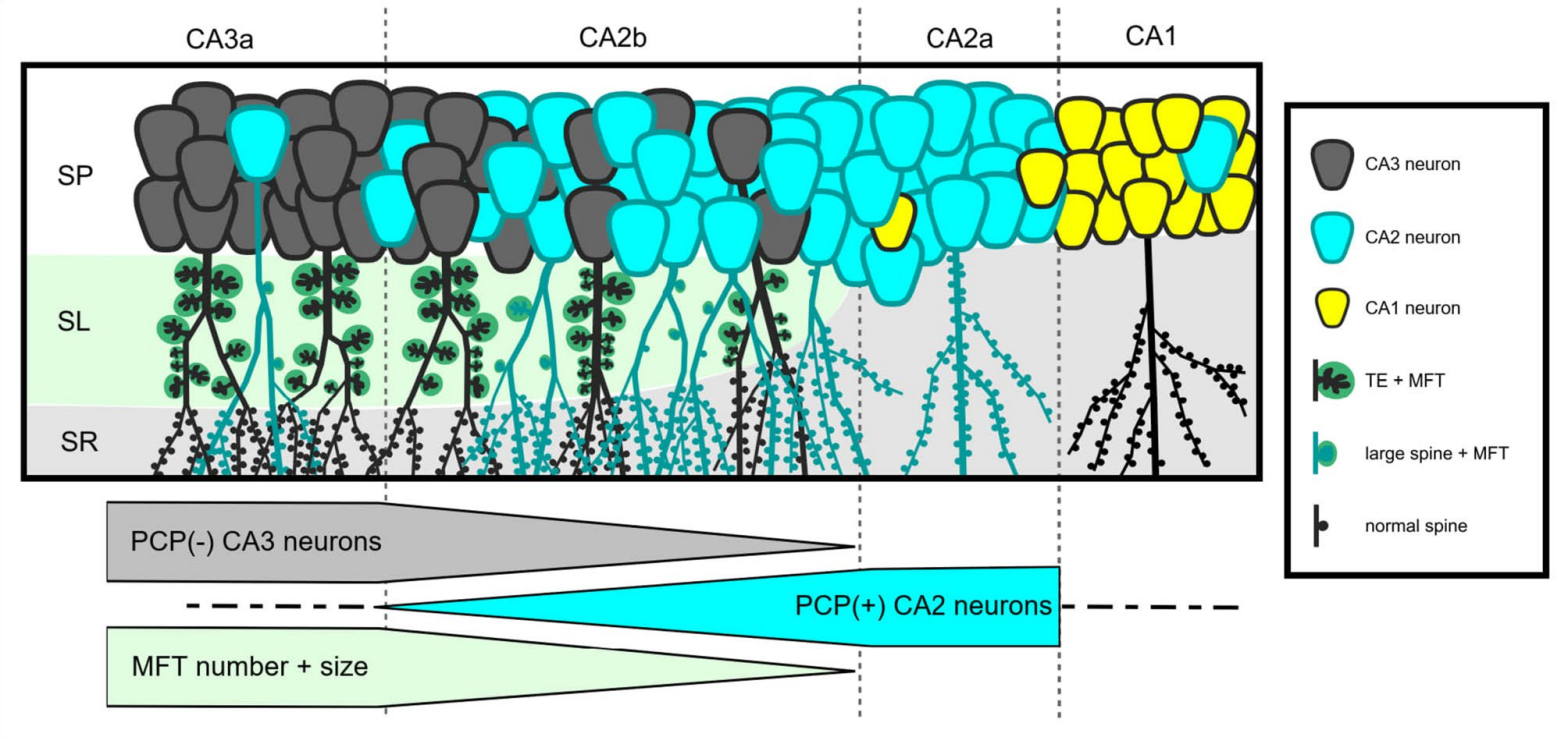
CA2b is a gradual transition zone between CA3 and CA2. Schematic diagram of pyramidal cell types along the dorsal CA3 – CA2 – CA1 axis. CA3a is populated by PCP4-negative CA3 neurons with large and numerous TEs. Mossy fiber terminals (MFT) are abundant and large in stratum lucidum (SL). Within CA2b, PCP4-negative CA3 neurons are gradually replaced by PCP4-positive CA2 neurons. Along their proximal apical dendrites, these PCP4-positive neurons display variable numbers of spines, larger spiny protrusions, and, rarely, TEs. As MFTs contacting PCP4-positive neurons are smaller and less numerous than the ones contacting PCP4-negative neurons, the width of SL decreases towards CA2. Within CA2a, PCP4-positive CA2 neurons predominate. They display proximal apical dendrites covered with normal-sized spines. In contrast, CA1 neurons exhibit aspiny or sparsely spiny proximal apical dendrites. Image is not to scale. MFT – *mossy fiber terminal*; TE – *thorny excrescence*; SO *– stratum oriens*; SP – *stratum pyramidale*; SL – *stratum lucidum*; SR *– stratum radiatum*; CA – *cornu ammonis.*

Several studies in mice have shown that the average MFT area and MFT number both gradually increase from distal CA2b (where the end of *stratum lucidum*/mossy fibers marks the border between CA2b and CA2a) towards CA3^7,9,14^. Our morphological data align well with these observations, and offer a plausible morphological explanation (cf. Figure 6). Distal CA2b is to a large extent composed of PCP4-positive CA2 cells which bear no, or only a few, larger spines that are contacted by MFTs, and only some CA3 cells with thorny excrescences are present. Accordingly, MFT numbers are lower due to the scarcity of postsynaptic partners, and the average MFT size is smaller due to the smaller size and complexity of the spines on CA2 neurons compared to thorny excrescences on CA3 neurons. Moving towards the proximal part of CA2b, more and more CA3 cells with regular thorny excrescences intermingle with CA2 cells, and an increasing number of CA2 cells carry at least some large spines on their proximal apical tree, accounting for the gradual increase of average MFT size and number in this area. Around the border of CA3a/CA2b, CA3 cells with thorny excrescences become the prominent cell type, explaining the rapid increase of large MFTs observed at around 200–300 μm proximal to the distal tip of *stratum lucidum*^9^.

Correctly and exclusively targeting CA2 neurons for morphologic analysis is a difficult task due to the small size of area CA2 and the lack of clear-cut borders. Approaches to specifically label CA2 cells found in the literature include diolistic labeling of the CA2 region combined with CA2 marker immunostaining^7^, the stereotactic injection of AAVs encoding fluorescent molecules directly into CA2, with or without CA2-specific promoters^9,27^, the use of transgenic mice expressing a fluorescent molecule under the control of CA2-specific promoters^1,23^, or a combination of the latter two, using stereotactic injection of floxed expression constructs into transgenic mice expressing Cre-recombinase selectively in CA2^28^. While these are elegant approaches to label CA2 as a whole, they do, however, lack single-cell resolution. As many or all cells in the targeted regions are labeled, a densely labeled dendritic plexus complicates the identification of single cells and their associated dendrites. It must also be considered that even the cell population targeted by largely CA2-specific promoters does not overlap fully with the cell population labeled by CA2 marker immunohistochemistry^9,27^. In addition, CA2b contains a mixture of CA3 and CA2 cells, and although not frequent, CA3 cells without thorny excrescences^25^ as well as CA2 cells with excrescence-like structures (this study) can be found in CA2b. As this implies that not every cell without thorny excrescences in this region is necessarily a CA2 cell, and vice versa not every neuron displaying such structures is a CA3 cell, every non-celltype-specific labeling approach of neurons in this region should include a post-hoc immunohistochemical verification of CA2 identity. While electrophysiologists have the advantage of obtaining single-cell morphologies by filling the neurons they record from with a fluorescent dye, and can post-hoc stain for a CA2 marker, that approach is time consuming, and requires specialized and expensive equipment. The strength of our approach lies in the sampling of a large number of single cells in adult, naïve wildtype animals, largely unconfounded by neighboring cells and dendrites. In combination with the identification of CA2 and non-CA2 cells using PCP4 immunohistochemistry, it gives a comprehensible overview of the relative cell type distributions in the murine wildtype dorsal area CA2. In conclusion, we observed a transitional gradient from CA3 neurons with thorny excrescences to largely athorny CA2 neurons within CA2b. Thus, CA2b is a hybrid hippocampal area in which CA3 and CA2 neurons intermingle and receive cell-specific input. Cellular analyses of identified cells will be of the essence to understand the function of this subregion in social learning.

## Materials and methods

### Animals

For the light microscopic study, data were obtained from five male and six female C57BL/6 J mice aged between 10 and 14 weeks. All analyzed sections had been prepared in the course of a previous study^19^ and were reimaged and reevaluated for this study. Two additional male mice (16 weeks) were used for the EM part of the study. Animals were bought from Janvier (Janvier Labs, France) and housed at the animal facility of the Goethe-University Hospital Frankfurt with water and food ad libitum and a 12 h/12 h light-dark-cycle until they were perfused. Perfusion experiments were conducted in accordance with the German Animal Welfare Act and approved and licensed by the responsible government body (Regierungspräsidium Darmstadt; FU/2039). Every effort was made to minimize distress and pain of the animals. Methods are reported in accordance with the ARRIVE guidelines.

### Iontophoretic filling of pyramidal neurons

Fillings were done as described previously^19^. Shortly, mice were transcardially perfused with 0.1 M phosphate-buffered saline (PBS), then fixed with 4% paraformaldehyde (PFA) in 0.1 M PBS for 5 min. The brain was postfixed for 18 h at 4 °C in the same fixative. After three washes in ice-cold 0.1 M PBS, frontal slices of 250 μm thickness were cut on a vibratome (Leica VT 1000 S) using a custom-built object holder to tilt the brain in an approx. 30° angle to account for the branching of CA2 neurons along the transverse axis of the hippocampus. Sharp quartz-glass microelectrodes (Sutter Instruments, QF100-70-10, with filament) were pulled using a P-2000 laser puller (Sutter Instruments). Microelectrodes were loaded with 0.5 mM Alexa Fluor 568 Hydrazide (Invitrogen), or in some cases 0.5 mM Alexa Fluor 488 (Invitrogen), in HPLC-grade water, and then back-filled with 0.1 M LiCl in HPLC-grade water. Sections containing the dorsal hippocampus were incubated for 10 min in the nuclear stain Hoechst diluted 1:5000 in 0.1 M PBS, then washed twice for 5 min in 0.1 M PBS. Sections were injected in a PBS-filled chamber on a fixed stage Olympus BX51 WI upright microscope placed on an x-y translation table (Science Products). Using a motorized 3D micromanipulator, the electrode was lowered into the slice under visual control while applying a negative voltage pulse (−1 V, 1 Hz) to the electrode. When piercing of a cell body was observed, the cell was filled by application of a negative 1 Hz current pulse to the electrode. Filling was done for 10 min or until no further filling was observed (Fig. 1A). Afterwards, slices were fixed for one to two days in 4% PFA at 4 °C and washed three times in cold 0.1 M PBS.

### Post-hoc immunohistochemistry

Post-hoc immunohistochemistry was done as described previously^19^. Filled and fixed injected Sect. (250 μm) were embedded in 5% agar and resliced to 40 μm-thin sections on a vibratome (Leica VT 1000 S) to facilitate staining. Free-floating sections were washed 3 times in 50 mM Tris-buffered saline (TBS) containing 0.1% Triton X-100, incubated in a blocking buffer (0.5% Triton X-100, 5% bovine serum albumin (BSA) in 50 mM TBS) for 30 min at room temperature (RT) and subsequently incubated with rabbit anti-PCP4 antibody (1:1000; Sigma-Aldrich Cat# HPA005792, RRID: AB_1855086. Species reactivity: mouse, human. Validated by the Human Protein Atlas project, see https://www.proteinatlas.org/ENSG00000183036-PCP4/summary/antibody) diluted in 0.1% Triton X-100 + 1% BSA in 50 mM TBS for 3 days at RT. After 4 washing steps, sections were incubated with goat anti-rabbit Alexa Fluor 647 secondary antibody (1:1000, Thermo Fisher Scientific Cat# A-21245, RRID: AB_2535813) for 4 h at RT, washed 4 times in 50 mM TBS, and mounted on glass slides with Dako fluorescence mounting medium. PCP4 staining was consistent with published data^1,7,9^.

### Imaging and analysis

Images were obtained on a Nikon C2 laser scanning confocal microscope. Laser lines 488, 568 and 633 were used to visualize Alexa 488 filling, Alexa 568 filling, and PCP4 staining, respectively. Overview image stacks of the injected region were taken using a 20x objective (Plan Apo, NA 0.75, Nikon), 1024 × 1024 resolution, 2x average and 1 μm z-size. Closeup images of individual cell bodies and dendrites were taken using a 60x oil immersion objective (Plan Apo VC, NA 1.40, Nikon), 1024 × 1024 resolution, and 0.5 μm z-size. For illustration purposes in the figures, individual segments were imaged with an additional digital zoom. The whole thickness of the slice was imaged. If cell bodies and proximal apical trees reaching into *stratum lucidum* were not on the same resliced section, the adjacent slice was imaged as well and dendritic trees were post-hoc assigned to their respective cell bodies (cf. Figure 1b). Filled cells were first sorted into the categories CA3a, CA2a, CA2b, CA1 based on the position of their cell bodies in the overview images (cf. Figure 1b). Borders were defined as follows: CA1/CA2a: onset of dense somatic and dendritic PCP4 labeling of pyramidal cells. CA2a/CA2b: distal end of *stratum lucidum*, CA2b/CA3a: end of dense labeling of somata with PCP4/start of a scarce and scattered labeling. While in many cases the CA2b/CA3 border could be drawn using the aforementioned criterion, staining of cell bodies at this border could also be less abrupt, showing a more gradual decrease. In these cases, defining this border was subject to the experimenters, decision.

The reslicing procedure after postfixation filling greatly facilitates PCP4-staining, but also cuts the filled dendritic tree. Labeled neurons were included into the analysis if they fulfilled the following criteria: The cell body could be identified as PCP4-negative or PCP4-positive. The apical trunk reaching through *stratum lucidum* was on the same reslice as the cell body, or was on an adjacent reslice and could be assigned to a corresponding cell body based on relative position (applying to all cells). If only a small part of filled apical trunk was visible in *stratum lucidum*, but thorny excrescences could be readily detected, cells were included. If no thorny excrescences were visible, the cells were included if the majority of apical trunk could be followed through *stratum lucidum* (applying only to cells in CA3a and CA2b). Filled cells in CA1 were included if spines were present on the basal or apical dendrites, irrespective of the length of apical dendrite visible.

For spine measurements, high-magnification image stacks were collected using a 60x oil immersion objective (Plan Apo VC, NA 1.40, Nikon) with 5x digital zoom, 2x average, 0.25 μm z-step size, and a 1024 × 1024 resolution (pixel size: 0.041 μm). Spine length and maximal head width of individual spiny protrusions within *stratum lucidum* or *stratum radiatum* on apical dendrites of PCP4-positive neurons in CA2b were measured using FIJI^29^ (RRID: SCR_002285). Only spines protruding laterally from the dendritic shaft and exceeding a length of 0.2 μm were considered^30^. Measurements were exported to GraphPad Prism 6 for analysis and data visualization. Spines were regarded as “large” if they exceeded a length of 1.5 μm AND displayed a bulbous head that was wider than 0.8 μm, or if their head exceeded a width of 1.2 μm, irrespective of length (see also Supplementary Fig. S2). Spines were regarded as a thorny excrescence if they showed a lobular structure with a stem and multiple bulbous heads, but were not measured in terms of spine length and head width due to their complex structure.

Maximal cross-sectional soma areas were measured in FIJI using the polygon selection tool to circle the filled cell body, and the “Measure” tool using “Area” as readout. For three of the 90 cells that were included into the distribution analysis, soma area could not be assessed. One cell body was cut directly at the surface of the slice, the other two had partially overlapping cell bodies.

### Electron microscopy

Mice were killed with an overdose of pentobarbital (500 mg/kg; Narcoren) and transcardially perfused with 0.9 M NaCl, then fixed with 4% PFA + 0,5% glutaraldehyde (GA) in 0.1 M cacodylate buffer for 10 min. Brains were removed and postfixed overnight in the same fixative. The next day, brains were washed in Tris-buffered saline (TBS), and serial 50 μm frontal brain sections were cut on a vibratome (Leica VT 1000 S) using the same custom-built object holder used for fillings. Sections containing the dorsal hippocampus were blocked with 5% bovine serum albumin (BSA) in TBS for 1 h at RT to reduce nonspecific staining. Sections were incubated for 20 h at RT with rabbit anti-PCP4 (1:500; Sigma-Aldrich Cat# HPA005792, RRID: AB_1855086) diluted in TBS + 2% BSA. After 4 washes in TBS, they were incubated for 90 min at RT with biotinylated goat anti-rabbit IgG (1:200; Vector Laboratories Cat# BA-1000, RRID: AB_2313606) diluted in TBS + 2% BSA. After washing 4 times in TBS, sections were incubated in avidin–biotin–peroxidase complex (ABC-Elite; Vector Laboratories) for 120 min at RT and were reacted with diaminobenzidine (DAB) solution (Vector Laboratories) containing 1% Cobalt chloride and 1% Ammonium nickel sulfate for 2–15 min at RT. After staining, sections were washed twice in TBS, then twice in 0.1 M cacodylate buffer for 5 min, osmicated (1% OsO4 in cacodylate buffer) for 30 min, dehydrated through an ascending ethanol series (30%, 50%, 1% uranyl acetate [Serva] in 70%, 80%, 90%, 100%, 100%), and finally embedded in Durcupan (Sigma-Aldrich). Sections were collected on single-slot Formvar-coated copper grids that were contrast-enhanced with lead citrate for 4 min, and examined using a Zeiss electron microscope (Zeiss EM 900). The CA2a/b border was set at the distal tip of stratum lucidum, which could be clearly distinguished by the presence (CA2b) or absence (CA2a) of MFTs and excrescence-like structures.

### Statistical analysis

Soma area data were analysed and visualized using GraphPad Prism 6. Averaged data are presented as Whisker-Dot-Plot, with upper/lower whisker representing maximum/minimum values, box representing median with upper and lower quartile, cross representing mean. The Kruskal-Wallis test was used to compare averaged data of CA3/PCP4-negative (*n* = 18), CA2/PCP4-negative (*n* = 14), CA2/PCP4-positive (*n* = 39), and CA1/PCP4 negative (*n* = 14) cells. In case of significance (*P* < 0.05), Dunn’s post-hoc comparison was employed. Adjusted p-values are reported. Corresponding statistical values in the text are expressed as mean ± standard deviation (s.d.). The two single CA3/PCP4(+) and CA1/PCP4(+) cells are presented in the graph of Fig. 4C for the sake of completeness, but were not included into the statistical analysis.

## Electronic supplementary material

Below is the link to the electronic supplementary material.

Supplementary Material 1

## Data Availability

Data is available from the corresponding author upon reasonable request.
