# Supplementary material for: Gradual transition of pyramidal cell types in the dorsal hippocampal area CA2b of the C57BL/6 mouse
Source: Sci Rep. 2025 Aug 11;15:29345. doi: 10.1038/s41598-025-04329-1 (PMC12340155; doi:10.1038/s41598-025-04329-1)
Supplement: Supplementary file 1 — Supplementary Material 1 [file 41598_2025_4329_MOESM1_ESM.pdf]

Supplementary information

"Gradual transition of pyramidal cell types in the dorsal hippocampal area CA2b of the C57BL/6 mouse"

Meike Fellenz, Rebecca Schneider, Sharif Jabra, Michael Rietsche, Dinko Smilovic, Mario Vuksic, David A. Slattery, and Thomas Deller

Content:

Supplementary Figure S1
Supplementary Figure S2, related to Figure 4
Supplementary Figure S3, related to Figure 4
Supplementary references

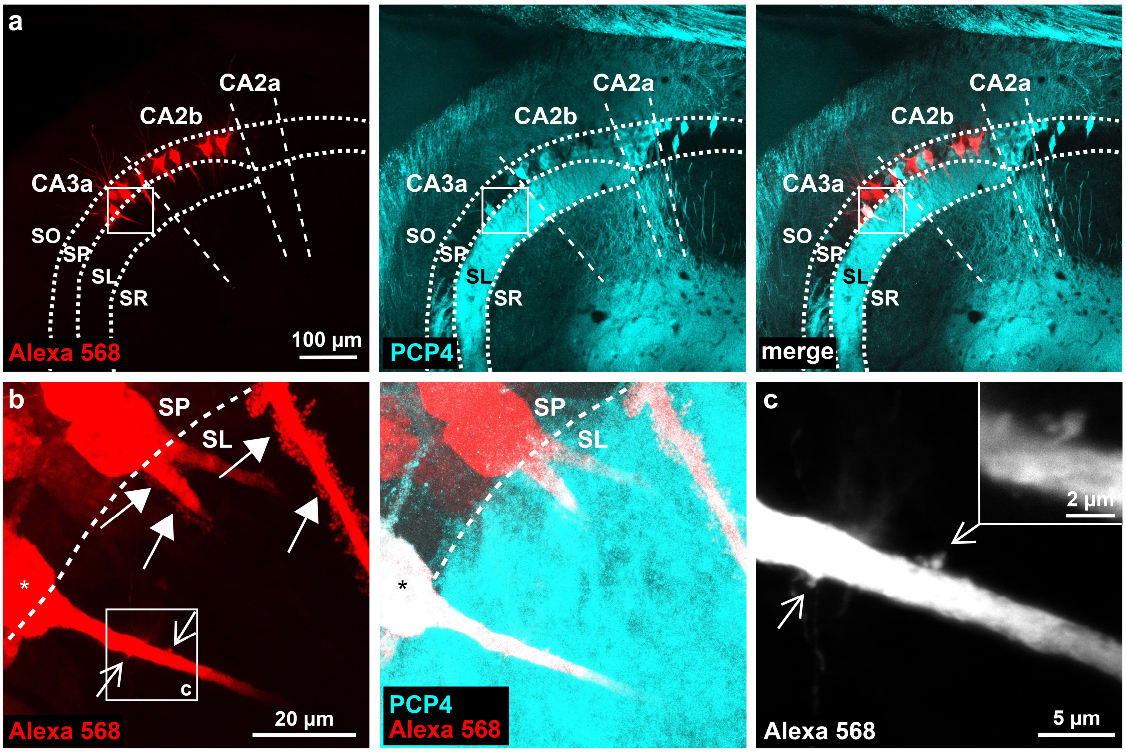

Supplementary Figure S1. PCP4-positive neuron in area CA3a

(**a**) Overview maximum Z projection of the injected CA region showing the relative position of a PCP4-positive neuron in CA3a. White box indicates the region magnified in **b**. (**b**) Magnification of the proximal apical trees show a mostly aspiny dendrite with sparse spineous structures (open arrows) on the PCP4-positive neuron (asterisk, compare PCP4-staining), while the PCP4-negative CA3 neurons in its vicinity are richly decorated with thorny excrescences (closed arrows). White box indicates the region magnified in **c**. (**c**) Closeup of the two spineous structures. SO – *stratum oriens*; SP – *stratum pyramidale*; SL – *stratum lucidum*; SR – *stratum radiatum*; CA – *cornu ammonis.*

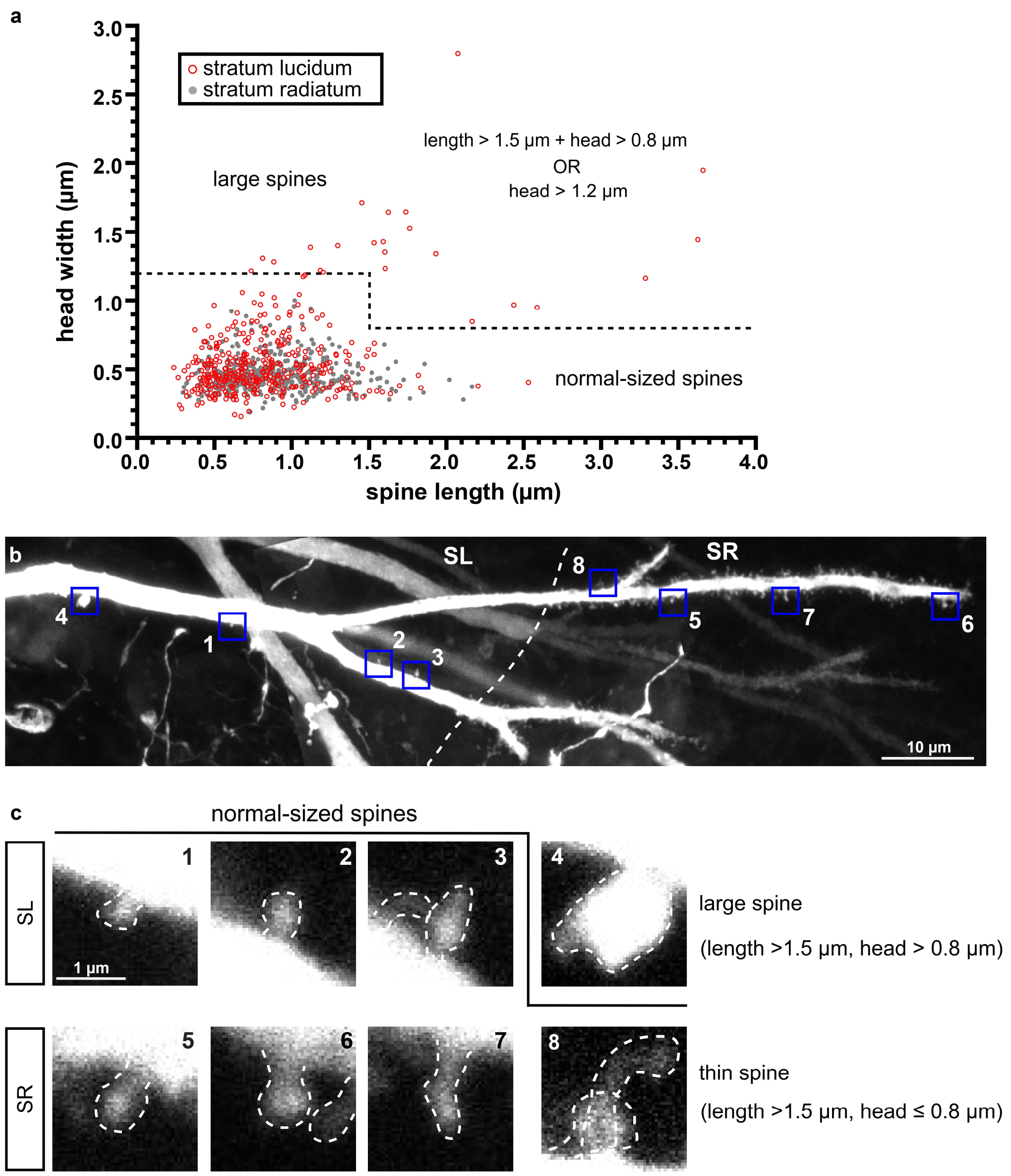

Supplementary Figure S2. Large spines are a distinct spine population on PCP4-positive CA2b neurons

(**a**) Scatter dot plot showing spine length and head width of individual spines on apical dendrites of PCP4-positive cells in stratum lucidum (SL, red dots) or stratum radiatum (SR, grey dots). SR: 465 spines from three PCP4-positive CA2b neurons of three different animals. SL: 383 spines from 26 PCP4-positive neurons in CA2b. Head widths of spines in SR did not exceed 1.0 µm. SR spines that were longer than 1.5 µm were rare, and only presented with small to medium-sized heads that did not exceed a width of 0.7 µm, reflecting the morphology of a filopodium or a thin spine. In contrast, SL dendrites, in addition to the normal-sized spines, displayed a second population of large spines that were considerably longer and/or had a larger head. Accordingly, spines were considered as "large" if they were longer that 1.5 µm and their head exceeded a width of 0.8 µm, or if the head width was larger than 1.2 µm, irrespective of length. Dotted line represents cut-off between normal-sized and large spines. (**b**) Stitched maximum-Z-projection of a PCP4-positive neuron in CA2b. Individual spines shown in (c) are marked by numbered blue boxes. (**c**) Higher magnification of spines on the apical dendrite of the CA2b neuron shown in (b). Spines that fall within the category of "normal-sized" have a comparable morphology in SL (1-3) and SR (5-7). Large spines (4) display a large, bulbous head, while normal spines of comparable length (8) only have a small head. Scale bar in c1 applies to c1-c8.

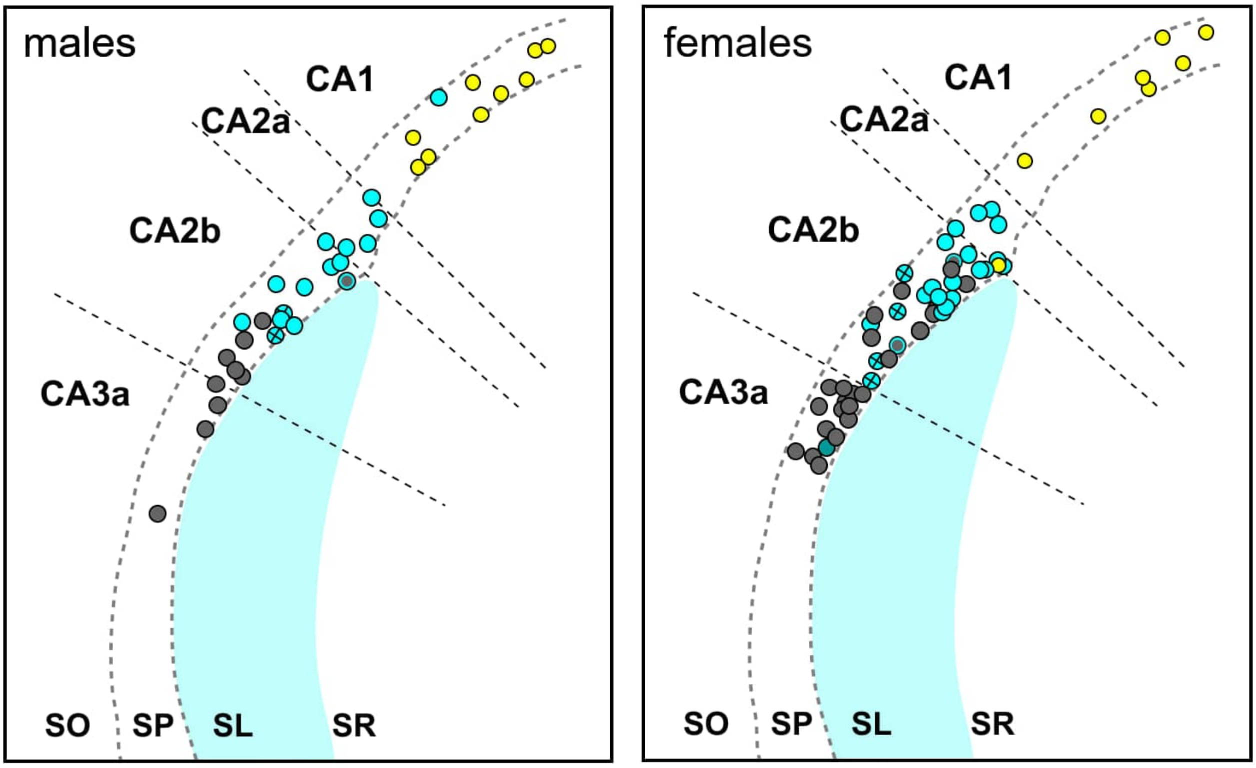

Supplementary Figure S2. No obvious differences between the sexes were detectable regarding cell distribution or spine morphology on the proximal apical dendrites in CA2b

PCP4-positive cells with thorny excrescences as well as PCP4-positive cells with large spines could be found in both sexes, and although cell numbers were lower for males than for females, intermingling of CA3 and CA2 cells in CA2b was observable in both males and females. The PCP4-positive cell in CA3a as well as the PCP4-negative CA1 cell in CA2a were both found in female animals. Yet, PCP4-positive stragglers in CA3a have also been found in male mice in another study[S1], and intermingling of CA2 and CA1 cells has also been reported in male rats[S2,S3]. It is therefore unlikely that the location of these cells is sex-specific.

Supplementary references

S1. Ding, L. *et al.* Structural Correlates of CA2 and CA3 Pyramidal Cell Activity in Freely-Moving Mice. *J. Neurosci. Off. J. Soc. Neurosci.* **40**, 5797–5806 (2020).

S2. Fernandez-Lamo, I. *et al.* Proximodistal Organization of the CA2 Hippocampal Area. *Cell Rep.* **26**, 1734-1746.e6 (2019).

S3. Ishizuka, N., Cowan, W. M. & Amaral, D. G. A quantitative analysis of the dendritic organization of pyramidal cells in the rat hippocampus. *J. Comp. Neurol.* **362**, 17–45 (1995).
